# The glycosyltransferase ALG3 is an AKT substrate that regulates protein N-glycosylation

**DOI:** 10.1016/j.jbc.2025.110582

**Published:** 2025-08-09

**Authors:** Adrija J. Navarro-Traxler, Laura Ghisolfi, Evan C. Lien, Alex Toker

**Affiliations:** Department of Pathology and Cancer Center, Beth Israel Deaconess Medical Center, Harvard Medical School, Boston, Massachusetts, USA

**Keywords:** PI3-kinase, AKT, signaling, N-glycosylation, ALG3, phosphorylation, glycans

## Abstract

AKT phosphorylates the glycosyltransferase ALG3. The PI3K/AKT signaling pathway is frequently dysregulated in cancer and controls key cellular processes such as survival, proliferation, metabolism, and growth. Protein glycosylation is essential for proper protein folding and is also often deregulated in cancer. Cancer cells depend on increased protein folding to sustain oncogene-driven proliferation rates. The N-glycosyltransferase asparagine-linked glycosylation 3 homolog (ALG3), a rate-limiting enzyme during glycan biosynthesis, catalyzes the addition of the first mannose to glycans in an alpha-1,3 linkage. Here we show that ALG3 is phosphorylated downstream of the PI3K/AKT pathway in both growth factor-stimulated cells and PI3K/AKT-hyperactive cancer cells. AKT directly phosphorylates ALG3 in the amino terminal region at Ser11/Ser13. CRISPR/Cas9-mediated depletion of ALG3 leads to improper glycan formation and induction of endoplasmic reticulum stress, the unfolded protein response, and impaired cell proliferation. Phosphorylation of ALG3 at Ser11/Ser13 is required for glycosylation of cell surface receptors EGFR, HER3, and E-cadherin. These findings provide a direct link between PI3K/AKT signaling and protein glycosylation in cancer cells.

The phosphoinositide 3-kinase (PI3K)/AKT pathway is a critical signaling network that controls cell growth, proliferation, metabolism, and survival. In response to extracellular stimuli including growth factors and hormones, activation of PI3K leads to synthesis of PIP3 and PI(3,4)P2, lipid second messengers that recruit downstream effectors to promote signal relay (1, 2, 3). Of these, the Ser/Thr protein kinase AKT is a major transducer of the PI3K signal that regulates a diverse array of substrates including protein and lipid kinases, transcription factors, small G proteins, metabolic enzymes, E3 ubiquitin ligases, and cell cycle regulators (1). AKT is a basophilic-directed protein kinase that phosphorylates Ser/Thr residues within a defined consensus motif of RXRXXs/t-Φ (where X is any amino acid, and Φ is any bulky hydrophobic residue) (4). Over 200 AKT substrates that harbor this consensus motif have been identified, whereby phosphorylation regulates enzymatic activity, cellular localization, and protein-protein interactions, leading to functional consequences in normal physiology and pathophysiology (1, 4, 5).

PI3K/AKT is one of the most frequently genetically dysregulated pathways in human cancers, due to somatic mutations and amplification of oncogenes such as *PIK3CA* (encoding the p110α catalytic subunit of class I PI3K) and *AKT1*, as well as inactivation of tumor suppressors such as phosphatase and tensin homolog (*PTEN*) and inositol polyphosphate 4-phosphatase type II (*INPP4B*) (1, 6). Mechanistically, PI3K/AKT signaling modulates cellular anabolic metabolism through the phosphorylation and regulation of rate-limiting metabolic enzymes involved in nucleotide, protein, and lipid biosynthesis (7). PI3K/AKT also controls glucose and glycogen metabolism, influencing glycolysis at the transcriptional and posttranslational level (8, 9, 10, 11). By contrast, a major role for PI3K/AKT signaling in the control of carbohydrate metabolism has not been described (12). Glycosylation, the process by which complex carbohydrates, or glycans, are covalently added to proteins, sugars, and lipids, is required for all aspects of normal cellular physiology and homeostasis (13, 14, 15). N-glycosylation, in which glycans are added to asparagine residues, is functionally critical for cell-surface receptors, secreted proteins, lysosomal enzymes, immunoglobulins, tumor antigens, and integrins (13, 14, 16, 17, 18, 19, 20, 21). N-glycosylation is altered during the metastatic transition in cancer, the epithelial to mesenchymal transition, and during antitumor immune responses (22, 23). Moreover, genes whose protein products participate in glycan synthesis, degradation, and modification are dysregulated in cancer, leading to aberrant glycan synthesis, or incomplete or truncated glycan structures (18, 24, 25, 26, 27, 28, 29, 30, 31, 32, 33, 34).

N-glycosylation is initiated in the endoplasmic reticulum (ER) and is necessary for proper protein folding and ER homeostasis (35, 36, 37). In the ER, glycosyltransferases catalyze the addition of specific carbohydrate moieties to a nascent glycan chain, with dolichol serving as a lipid carrier (12, 36, 37, 38, 39). Of these, the asparagine-linked glycosylation 3 homolog alpha-1,3- mannosyltransferase (ALG3), catalyzes the addition of the first Dol-P-Man derived mannose to man5GlcNAc2-PP-Dol in an alpha-1,3 linkage (40, 41).

In humans, compound heterozygous mutations in *ALG3* lead to congenital disorders of N-linked glycosylation (ALG3-CDG) (42, 43, 44, 45, 46). Patients with this rare disorder display severe neurological dysfunction, developmental delays, intellectual disabilities, and failure to thrive (42, 43, 44, 45, 46). Homozygous mutations in *ALG3* are rare, suggesting these may be lethal during embryonic development (42). Depletion of ALG3 in cells leads to improper glycan formation and induction of ER stress (45, 47, 48). In human cancers, *ALG3* is amplified in a variety of lineages, including lung, breast, ovarian, and esophageal cancers (49, 50, 51). Studies have also indicated that ALG3 expression is correlated with poor outcomes in breast cancer (50, 51). Here, we report that ALG3 is a target of PI3K signaling and is directly phosphorylated by AKT. Depletion of ALG3 induces ER stress and the unfolded protein response (UPR) with a concomitant deregulation of glycoproteins and reduced cell proliferation.

## Results

### ALG3 is an AKT substrate downstream of PI3K

**Figure 1:**
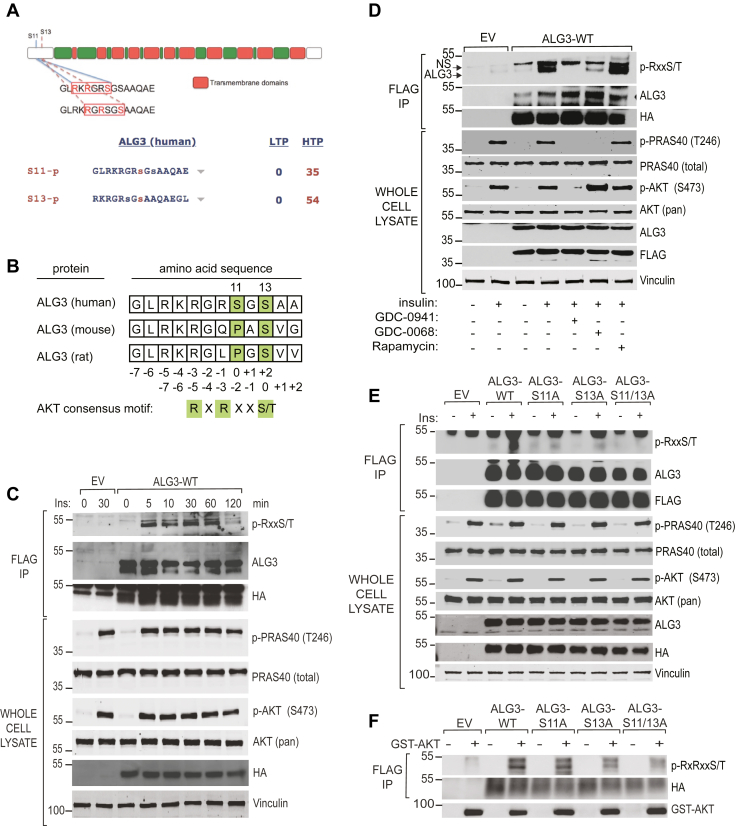
**ALG3 is an AKT substrate.***A*, ALG3 contains the AKT motif RXRXX-S/T at Ser11 and Ser13. *B*, ALG3 phosphosite conservation in mammals. Serum-starved MCF10A cells expressing EV or ALG3-WT (*C*) treated with 100 nM insulin (*D*) in the presence and absence of 15 min inhibitor pretreatment: 2 μM GDC-0941, 2 μM GDC-0068, or 20 nM rapamycin. NS denotes a nonspecific band. *Arrow* denotes ALG3. *E*, serum-starved MCF10A expressing EV, ALG3-WT, ALG3-S11A, ALG3-S13A, or ALG3-S11/13A treated with 100 nM insulin. *F*, EV, ALG3-WT, ALG3-S11A, ALG3-S13A, and ALG3-S11/13A (containing an additional 11 amino acids appended to the C terminus of ALG3) immunoprecipitated from serum starved MCF10A cells and incubated with active GST-AKT1 for 30 min. ALG3, asparagine-linked glycosylation 3; EV, empty vector.

To determine if glycosyltransferases represent a distinct class of AKT downstream targets, we screened the PhosphoSitePlus database (www.phosphosite.org) for proteins that have been mapped by global mass spectrometry phosphoproteomic studies and which contain the minimal AKT consensus motif RXRXXs/t (52). This analysis revealed a number of glycosyltransferases which harbor posttranslational modifications including Ser/Thr/Tyr phosphorylation, Lys/Arg mono-methylation, Lys ubiquitylation, Lys acetylation, Lys succinylation, Asn N-glycosylation, Ser/Thr O-GlcNAc, and Arg di-methylation. Within this group, ALG3 contains two high-quality basophilic phosphorylation motifs at Ser11/Ser13 that conform to the optimal AKT consensus motif (Fig. 1*A*) (5). Phosphorylation of ALG3 at Ser11/Ser13 has been detected in multiple independent high throughput proteomic studies (Fig. 1*A*). The Ser11/Ser13 consensus motifs are conserved in mammalian species, including human, mouse, and rat (Fig. 1*B*). To determine if ALG3 is phosphorylated downstream of PI3K/AKT, HA/FLAG-tagged ALG3 (ALG3-WT) was expressed in MCF10A cells, an immortalized mammary epithelial cell line that responds robustly to growth factor stimulation and subsequent PI3K/AKT activation. Cells were serum-starved, stimulated with insulin over a time course, ALG3 immunoprecipitated, and phosphorylation detected with a phospho-specific antibody that recognizes the AKT consensus motif. Insulin stimulated ALG3 phosphorylation in a time-dependent manner, coincident with PI3K/AKT activation as measured by pAKT (Ser473) and the canonical substrate PRAS40 (Ser246) (Fig. 1*C*). ALG3 phosphorylation in response to insulin was blocked by GDC-0941 (pan-PI3K inhibitor), GDC-0068 (pan-AKT inhibitor), but not by rapamycin (MTORC1 inhibitor), suggesting that ALG3 phosphorylation occurs downstream of AKT, but not S6K (Fig. 1*D*). Furthermore, insulin-stimulated phosphorylation of ALG3 was not observed in individual ALG3.Ser11Ala (ALG3-S11A), ALG3.Ser13Ala (ALG3-S13A), and compound ALG3.Ser11Ala/Ser13Ala mutants (ALG3-S11/13A), compared to WT ALG3 (Fig. 1*E*). In an *in vitro* protein kinase assay, recombinant active GST-AKT1 phosphorylated immuno-isolated recombinant ALG3-WT, but not ALG3-S11A, ALG3-S13A or ALG3-S11/13A mutants (Fig. 1*F*). Collectively these data demonstrate that ALG3 is an AKT substrate downstream of PI3K, and is phosphorylated at Ser11/Ser13, in agreement with global phosphoproteomic analyses.

### ALG3 regulates protein N-glycosylation

**Figure 2:**
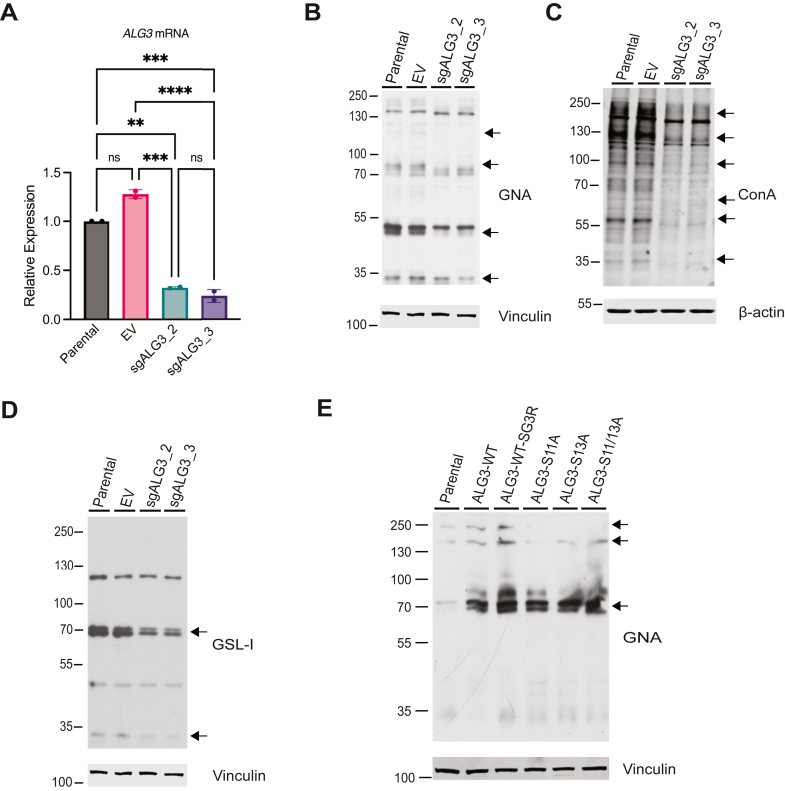
**ALG3 expression levels influence lectin binding.** MDA-MB-468 parental cells and cells expressing CRISPR EV or guides sgALG3_2 or sgALG3_3. *A*, *ALG3* mRNA abundance measured by qRT-PCR expressed as fold change relative to parental cells. Data are representative of seven biological replicates with two to four technical replicates. Statistical analysis was performed using two-way analysis of variance (ANOVA) with Tukey’s multiple comparisons test. sgALG3_2 and sgALG3_3, ∗∗*p* = 0.0019, ∗∗∗*p* = 0.008; and EV ∗∗∗*p* = 0.002, ∗∗∗∗*p* < 0.001. Lectin staining was performed using (*B*) GNA, (*C*) ConA, and (*D*) GSL-I. *E*, MCF10A parental cells and cells expressing ALG3-WT, ALG3-WT-SG3R, ALG3-S11A, ALG3-S13A, and ALG3-S11/13A stained for GNA. *Arrows* indicate proteins with differential lectin staining. The same vinculin loading control blot is shown for 2B and 2D as the samples originate from the same experiment. ALG3, asparagine-linked glycosylation 3; GNA, *Galanthus nivalis* agglutinin; ConA, concanavalin A; GSL-I, *Griffonia simplicifolia l*ectin I; EV, empty vector.

To determine whether ALG3 functionally influences protein N-glycosylation, lectin staining was used to profile whole cell lysates in MDA-MB-468 cells, a triple negative breast cancer cell line with *PTEN* inactivation that displays hyperactive AKT signaling. CRISPR/Cas9 pooled cell lines were generated using two independent guides for ALG3 (sgALG3_2 and sgALG3_3), and control empty vector. ALG3 KO was confirmed by quantitative reverse transcription-polymerase chain reaction due to the lack of an available antibody that robustly detects endogenous ALG3 (Fig. 2*A*). Cells were grown over a period of 8 days through selection, harvested, and whole cell lysates profiled by lectin staining using three distinct lectins that recognize glycans inclusive of α-1,3 mannose: *Galanthus nivalis* agglutinin (GNA), concanavalin A (ConA) and *Griffonia simplicifolia* lectin I (GSL-I). GNA binds hybrid glycans, specifically terminal α-1,3 mannose residues (15). ConA lectin recognizes α-mannose-containing cores and oligomannose-type N-glycans with a higher affinity than complex-type N-glycans, but not highly branched complex-type N-glycans nor O-glycans (15). GSL-I binds terminal Galα and GalNAcα (15).

CRISPR-Cas9 depletion of ALG3 reduced the staining pattern of all three lectins, indicating that ALG3 is functionally required for protein glycosylation using α-1,3 mannose in breast cancer cells with hyperactive PI3K signaling (Fig. 2, *B*–*D*). Specifically, decreased staining of bands migrating at 120 kDa, 80 kDa, 50 kDa, and 30 kDa was observed with GNA; 200 kDa, 120 kDa, 100 kDa, 60 kDa, 57 kDa, and 35 kDa with ConA; 70 kDa and 33 kDa with GSL-I. Ectopic expression of ALG3-WT in MCF10A cells increased the pattern of protein bands recognized by GNA lectin at 70 kDa, 150 kDa, and 240 kDa. Guide-resistant ALG3-WT, ALG3-S11A, ALG3-S13A, and ALG3-S11/13A were also expressed and similarly enhanced GNA staining of all three proteins, however the phospho-mutant alleles did not show enhanced staining of proteins at 150 kDa and 240 kDa (Fig. 2*E*). This suggests that phosphorylation of Ser11/Ser13 regulates the ability of ALG3 to modulate the glycome processed within the N-glycosylation pathway.

### ALG3 depletion induces ER stress and the UPR

**Figure 3:**
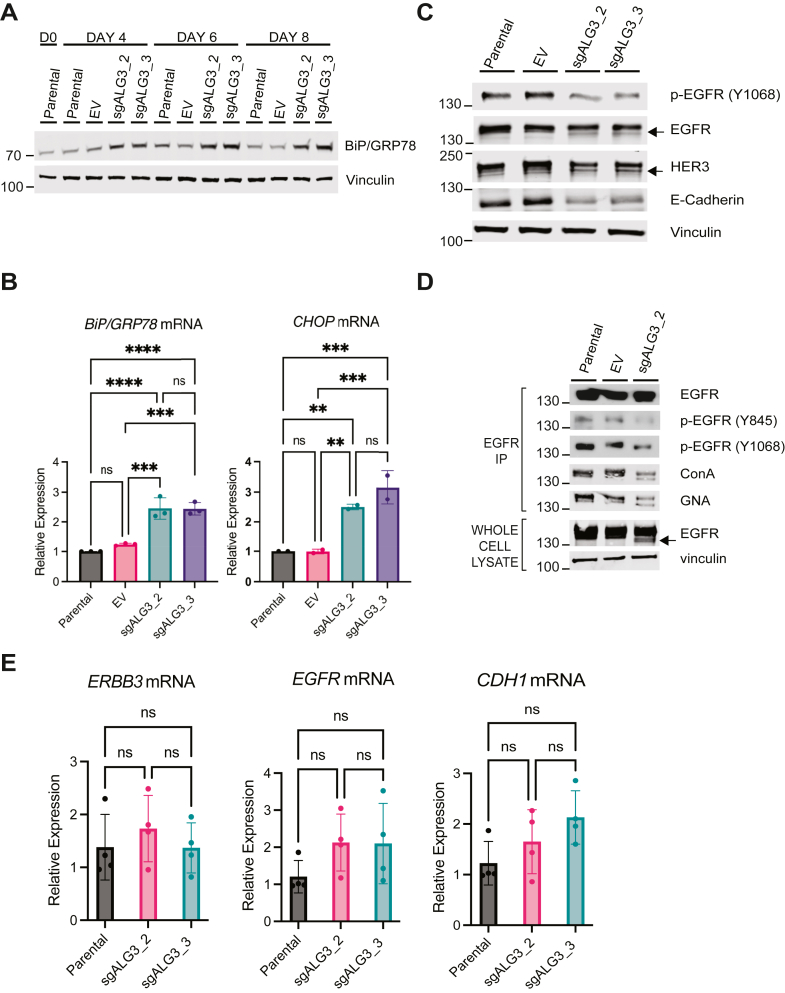
**ALG3 depletion induces ER stress and attenuates receptor glycosylation.***A*, whole cell lysates of MDA-MB-468 parental, EV, and sgALG3_2 and sgALG3_3 cells immunoblotted for BiP/GRP78 and (*B*) by qRT-PCR for *BiP/GRP78* mRNA and *CHOP* mRNA. The data are representative of three biological replicates with at least four technical replicates. mRNA expressed as fold change relative to the parental line and normalized to *GAPDH* mRNA and *18S* mRNA; *asterisks* (∗) indicate significant differences in *BiP/GRP78* mRNA levels for KO cells compared to parental (∗∗∗∗*p* < 0.0001) and EV cells (∗∗∗*p* = 0.0002) and indicate significant differences in *CHOP* mRNA levels for KO cells compared to parental (∗∗*p* = 0.0033; ∗∗∗, *p* = 0.0003) and EV cells (∗∗*p* = 0.0032; ∗∗∗*p* = 0.0003). *C*, MDA-MB-468 cells transduced with EV or sgALG3_2/3. *Arrows* indicate migration of deglycosylated receptors. *D*, EGFR immunoprecipitates and lysates from MDA-MB-468 and cells transduced with EV or sgALG3_3. *E*, qRT-PCR analysis of mRNA in MDA-MB-468 cells expressing EV or sgALG3_2/3. mRNA expressed as fold change relative to the parental line and normalized to *18S* mRNA. The data are representative of two biological replicates with two technical replicates. Statistical analysis was performed using one-way ANOVA with Tukey’s multiple comparisons test. ALG3, asparagine-linked glycosylation 3 homolog; CHOP, C/EBP homologous protein; EGFR, epidermal growth factor-receptor; ER, endoplasmic reticulum; EV, empty vector.

Protein glycosylation occurs in the ER and is both necessary and sufficient for proper protein folding and ER homeostasis. Enhanced N-glycosylation supports increased protein turnover and renewal required by rapidly proliferating cells (4, 13, 14, 35). By contrast, impaired N-glycosylation results in accumulation of unfolded proteins, leading to ER stress and activation of the UPR, or cell death when ER stress cannot be resolved (53). CRISPR-Cas9 depletion of ALG3 (sgALG3_2 and sgALG3_3) led to induction of ER stress in MDA-MB-468 breast cancer cells, as determined by increased protein (Fig. 3*A*) and mRNA (Fig. 3*B*) of the ER stress and UPR marker 78 kDA glucose-regulated protein/binding immunoglobulin protein (*GRP78/BiP*). Moreover, a corresponding increase in the mRNA levels of the ER/UPR marker C/EBP homologous protein (*CHOP*) was also observed upon ALG3 depletion (Fig. 3*B*).

To determine the functional consequence of ALG3 depletion on proper protein folding, we evaluated a number of integral membrane proteins known to be glycosylated, including epidermal growth factor-receptor (EGFR, 11 N-glycosylation sites), human epidermal growth factor receptor 3 (HER3, 10 N-glycosylation sites), and E-cadherin (4 N-glycosylation sites). Depletion of ALG3 in MDA-MB-468 cells resulted in reduced levels of phosphorylated EGFR and E-cadherin, indicative of improper protein folding. Moreover, for both EGFR and HER3, a faster migrating band was resolved by SDS PAGE indicative of deglycosylated receptors (Fig. 3*C*). Depletion of ALG3 in MDA-MB-468 cells also resulted in reduced ConA and GNA lectin staining of immunoprecipitated EGFR, concomitant with reduced tyrosine phosphorylation at pY845 and pY1068, suggestive of impaired receptor activation due to improper receptor glycosylation and folding (Fig. 3*D*). The mRNA levels of *EGFR*, *ERBB3* (HER3), and *CDH1* (E-cadherin) were unaffected by ALG3 depletion (Fig. 3*E*).

### ALG3 is functionally required for cell proliferation

**Figure 4:**
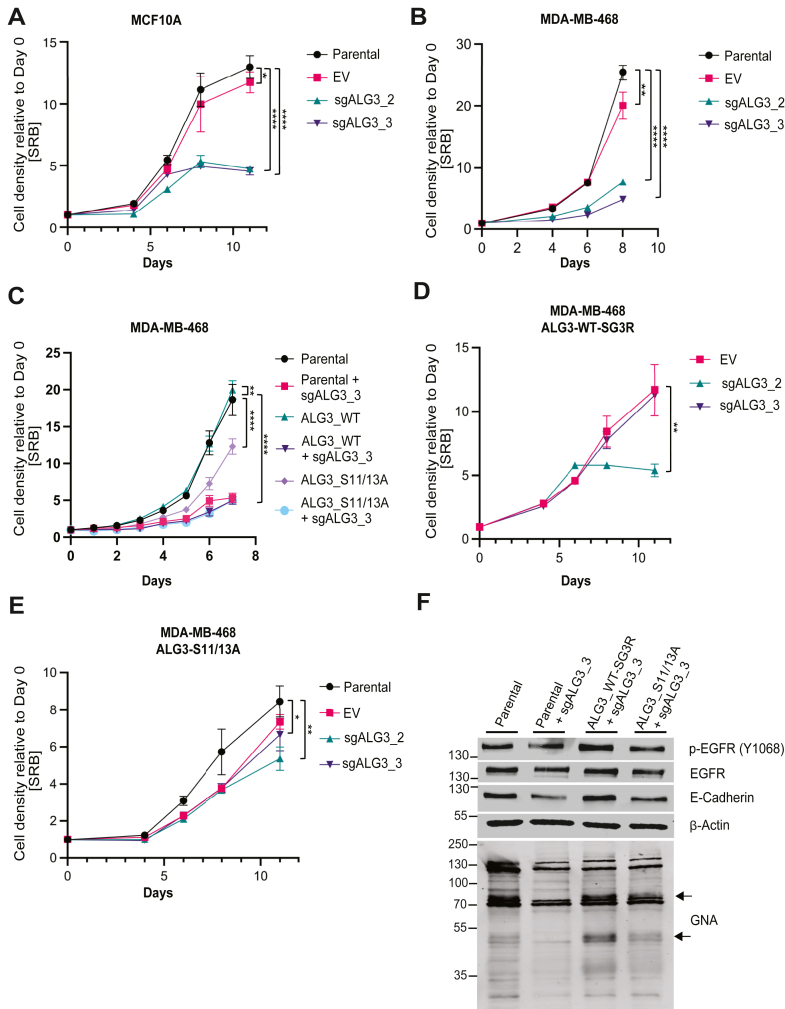
**Depletion of ALG3 reduces proliferation of MCF10A and MDA-MB-468 cells.***A*, SRB proliferation assay for parental cells and cells expressing EV or sgALG3_2/3 in MCF10A (∗*p* = 0.0381; ∗∗∗∗*p* < 0.0001) (data are representative of three biological replicates with three technical replicates) and (*B*) MDA-MB-468 cells (∗∗*p* = 0.0020; ∗∗∗∗*p* < 0.0001) (data are representative of six biological replicates with three technical replicates). *C*, SRB assay for MDA-MB-468 parental cells and cells overexpressing ALG3-WT or ALG3-S11/13A, infected with sgALG3_3 and respective parental counterparts (∗∗*p* = 0.0091; ∗∗∗∗*p* < 0.0001) (*D*) SRB for MDA-MB-468 cells expressing ALG3-WT-SG3R (resistant to guide sgALG3_3 but not resistant to sgALG3_2) transduced with EV or sgALG3_2 and sgALG3_3 (∗∗*p* = 0.0012). *E*, SRB for MDA-MB-468 cells expressing ALG3-S11/13A with EV or sgALG3_2 and sgALG3_3 (∗∗*p* = 0.0018; ∗, *p* = 0.0371). For *C*–*E*, data are representative of three biological replicates with at least three technical replicates. *F*, immunoblotting of MDA-MB-468 cells and parental, ALG3-WT-SG3R and ALG3-S11/13A-SG3R cells infected with sgALG3_3 for p-EGFR (pY1068), EGFR, E-Cadherin, β-actin, and GNA lectin. For *A*–*D*, data are expressed as mean ± SD. Data are representative of two biological replicates with three technical replicates. Statistical analysis performed using end point data, an ordinary one-way ANOVA and Dunnett’s multiple comparisons test. ALG3, asparagine-linked glycosylation 3 homolog; EGFR, epidermal growth factor-receptor; GNA, *Galanthus nivalis* agglutinin; EV, empty vector; SRB, sulforhodamine B.

We next determined whether ALG3 is required for cell proliferation in cells harboring hyperactivated AKT signaling. First, in WT MCF10A mammary epithelial cells grown in full serum, upon depletion of ALG3 *via* CRISPR knockout with two independent guides (sgALG3_2, sgALG3_3) cell proliferation was markedly attenuated compared to parental and control cells (Fig. 4*A*). Similarly, depletion of ALG3 also significantly reduced cell proliferation in *PTEN*-deficient, AKT-hyperactivated MDA-MB-468 breast cancer cells (Fig. 4*B*). Next, MDA-MB-468 parental cells and cells overexpressing WT and phosphomutant ALG3 were infected with sgALG3_3 then grown alongside their parental counterparts for 7 days. For all lines, depletion of ALG3 resulted in decreased proliferation. Although overexpression of ALG3-WT resulted in a modest increase in cell proliferation relative to parental cells, overexpression of ALG3-S11/13A resulted in decreased cell proliferation relative to parental cells, suggesting that Ser11/Ser13 phosphorylation is critical for cell growth (Fig. 4*C*). The ALG3-WT cDNA was subjected to site-directed mutagenesis to render it resistant to guide sgALG3_3, but not resistant to guide sgALG3_2 and is thus denoted as ALG3-WT-SG3R. Inhibition of proliferation in MDA-MB-468 cell transduced with CRISPR-Cas9 guides sgALG3_2 and sgALG3_3 was rescued with ALG3-WT-SG3R (Fig. 4*D*). Similarly, MDA-MB-468 cells harboring an SG3 guide-resistant ALG3 ser11ala/ser13ala phosphomutant (ALG3-S11/13A) also rescued proliferation, albeit to a lesser extent than to cells expressing guide-resistant WT ALG3 (in the context of vector-transduced cells compared to guide sgALG3_2; Fig. 4*E*). Finally, expression of ALG3-WT-SG3R in MDA-MB-468 cells transduced with sgALG3_3 rescued total EGFR protein levels, EGFR Y1068 phosphorylation and E-cadherin total protein, as well as the GNA staining pattern. By contrast, expression of sgALG3-resistant ALG3-S11/13A failed to rescue, suggesting that ALG3 phosphorylation at Ser11/Ser13 is required for ALG3 function in glycosylation and protein folding of these glycoproteins (Fig. 4*F*).

## Discussion

Both N-glycosylation and PI3K/AKT signaling are critical for normal proliferation and growth, and their deregulation contributes to tumorigenesis. Here, we show that growth factor and oncogene signaling through PI3K/AKT regulates N-glycosylation to support the increased protein folding needs of rapidly proliferating cancer cells. We show that ALG3 is phosphorylated at Ser11 and Ser13 by AKT in growth factor-stimulated cells, and that phosphorylation of ALG3 is required for proper glycan addition and protein folding of cell surface receptors. Although AKT typically inhibits substrates upon phosphorylation, it also upregulates anabolic pathways such as lipid, protein, and nucleotide metabolism to satisfy the needs of rapidly proliferating cells (1, 54). Our data are consistent with a model in which phosphorylation of ALG3 by AKT is an activating event, accelerating N-glycan production to meet the demands of oncogenic signaling. This is consistent with the finding that inhibition of ALG3 leads to induction of the unfolded protein response, ER stress, and impaired cell proliferation in PI3K/AKT hyperactive cancer cells. Impaired phosphorylation of ALG3 using Ser11/Ser13 phosphomutants only modestly affected cell proliferation, at least under our experimental conditions, indicating that other modifications may contribute to N-glycosylation and activity. By contrast, impaired ALG3 Ser11/Ser13 phosphorylation attenuated protein glycosylation and reduced expression of deglycosylated receptors such as EGFR and HER3.

Prior studies have suggested a functional role for ALG3 in malignancy. In *Drosophila*, ALG3 loss disrupts N-glycosylation of the tumor necrosis factor receptor, elevated c-Jun N-terminal kinase, suppression of the Hippo pathway and increased cell proliferation (55, 56). In humans, *ALG3* gene amplification, associated with poor outcomes in breast cancer, has also been observed in various other human cancers (50, 51, 57, 58). The *ALG3* gene resides on the same 3q26 to 27 amplicon as *PIK3CA*, which encodes the p110α catalytic subunit of class I PI3K. Consequently, *ALG3* and *PIK3CA* are frequently coamplified (57, 58). Moreover, while ALG3 overexpression promotes cancer progression, loss-of-function *ALG3* mutations result in severe clinical manifestations in congenital disorders of N-glycosylation (ALG3-CDG). These patients harbor compound heterozygous *ALG3* mutations, but homozygous mutations are rarely found, suggesting these may be lethal during embryonic development (42). ALG3-CDG patients display developmental and intellectual disabilities, muscular hypotonia, cerebral malformations, neurological features such as epileptic seizures, cardiac defects, facial and body dysmorphism, and metabolic dysfunction including refractory hypoglycemia (42, 44). Molecularly, ALG3-CDG patients have accumulated truncated Man5-GlCNAc2 structures, expected to be found in cells lacking functional ALG3 (46). Taken together, these findings underscore a critical balance in ALG3 expression and function.

We propose a model in which in addition to anabolic protein, nucleotide, and lipid metabolism, the PI3K/AKT pathway promotes N-glycosylation through ALG3 to facilitate proper folding of proteins destined for secretion and the cell surface in rapidly proliferating cells. Identifying the precise identity of the glycome and the glycoproteome regulated by PI3K/AKT/ALG3 is predicted to uncover a new layer on the role of PI3K/AKT in cancer. These findings also raise the question as to whether additional glycosyltransferases are also functionally modified by posttranslational modifications. *In silico* data mining reveals that, for example, α-1,3/1,6-mannosyltransferase (ALG2) also harbors an AKT substrate consensus that has been mapped at Ser256 (59). Similarly, a link between MEK-ERK signaling and N-glycosylation is suggested by the fact that the dolichol-phosphate mannosyltransferase subunit 1 (DPM1) harbors multiple putative proline-directed kinase (*e.g.* ERK and CDK) phosphorylation sites (59, 60). DPM1 catalyzes the first committed step in the N-glycosylation pathway and is also amplified in cancer (15, 61, 62). These represent just two examples of glycosyltransferases that are likely modified by Ser/Thr phosphorylation in cells and tissues, raising the possibility that functional modulation of the proteoglycome lies downstream of oncogene-driven signaling pathways.

Understanding the regulation of N-glycosylation by signaling pathways such as PI3K/AKT may also afford new therapeutic interventions in diseases such as cancer and congenital disorders of N-glycosylation. Deregulated glycosylation has been associated with a failure to thrive in infants, impaired neurological development, and infantile-onset symptomatic epilepsy (20, 63). Uncovering how signaling pathways regulate glycosylation and how aberrant signaling can alter glycosylation may provide new approaches for the combination strategies targeting both protein kinases and glycosylation. As but one example, drugs that inhibit oligosaccharyltransferase, the enzyme that transfers oligosaccharides to proteins to facilitate folding, have shown promise in non–small cell lung cancer by selectively blocking EGFR (64). Collectively, these findings advance our understanding of the regulation of N-glycosylation by growth factor and oncogene-driven signaling and its role in normal cellular physiology as well as cancer.

## Limitations of the study

Although our data point to a critical role for ALG3 phosphorylation by AKT and furthermore that this is an activating event, due to technical limitations we were unable to demonstrate this directly. Studies are needed to determine whether phosphorylation of ALG3 at Ser11/Ser13 alters glycosyltransferase activity, conformation, or interactions with partner proteins. Moreover, a comprehensive glycomics-driven survey of downstream glycoprotein substrates, alongside structural and biochemical analyses of the AKT-ALG3 interface, represent important next steps. Although our data suggest that phosphorylation of ALG3 may influence protein stability, we have not rigorously assessed protein turnover. Finally, although ALG3 regulation by AKT affects receptors such as EGFR, we have not directly determined protein folding or receptor activity.

## Experimental procedures

Detailed experimental procedures can be found in the supporting information.

## Quantification and statistical analysis

Statistical analyses were performed with PRISM 10 (GraphPad), and each data point represents the mean ± SD. The number of biological and technical replicates is listed in the supporting information. Statistical analyses for growth curves were performed with one-way ANOVA.

## Data availability

Source data and annotated analysis workflows are available on the following OSF project website: https://osf.io/v4tzs/.

## Supporting information

This article contains supporting information (65, 66, 67, 68, 69).

## Conflict of interest

A. T. is a consultant for Atavistik, Inc. and receives funding support from Myris Therapeutics. A. T. is the Editor-in-Chief of the Journal of Biological Chemistry. The other authors declare that they have no conflicts of interest with the contents of this article
