# Supplementary material for: The glycosyltransferase ALG3 is an AKT substrate that regulates protein N-glycosylation
Source: J Biol Chem. 2025 Aug 9;301(9):110582. doi: 10.1016/j.jbc.2025.110582 (PMC12451169; doi:10.1016/j.jbc.2025.110582)
Supplement: Supporting Information [file mmc1.docx]

## Supporting Information – Methods Description

Further information and requests for resources and reagents should be directed to and will be fulfilled by the lead contact, Alex Toker ([atoker@bidmc.harvard.edu](mailto:atoker@bidmc.harvard.edu)). Requested compounds will be provided following completion of an MTA.

**Cell Culture.**

MCF10A cells were cultured in standard MCF10A growth medium without antibiotics (DMEM/F12 medium (Wisent Bioproducts, 319-075-CL), 5% horse serum (Gemini Bio, 100508), 10 µg ml^-1^ insulin (Thermo Fisher Scientific/Gibco, A1138211), 0.5 mg ml^-1^ hydrocortisone (Sigma-Aldrich,H4001), 20 ng ml^-1^ EGF (R&D Systems, 236-EG-01M) and 100 ng ml^-1^ cholera toxin (List Biological Laboratories, 100B). All MDA-MB-468-derived cell lines were cultured in RPMI media (Wisent Bioproducts) supplemented with 10% heat-inactivated fetal bovine serum (Thermo Fisher Scientific) without antibiotics. All cell lines were cultures at 37°C in the presence of 5% CO_2_. Cells were passaged for no more than 4 months and routinely assayed for mycoplasma contamination.

**Inhibitors and Growth Factors**

Serum/growth-factor-deprived cells (20 hours) were pre-treated for 15 minutes with DMSO (Thermo Fisher Scientific, BP231-100) or small-molecule inhibitors, then treated with insulin (Thermo Fisher Scientific/Gibco, A1138SII [dissolved in 0.1N HCl then diluted in water to final concentration]) for the indicated time period. Stocks were prepared under sterile conditions, aliquoted and stored at –80C. The small-molecule kinase inhibitors used included the PI3K catalytic inhibitor GDC-0941 (Cayman Chemical, 11600), the AKT catalytic inhibitor GDC-0068 (Selleck Chemicals, S2808) which were each dissolved in DMSO at 10mM. The mTORC1 inhibitor Rapamycin (Cayman Chemical, 13346) was dissolved in DMSO at 100uM . All inhibitors were aliquoted under sterile conditions, and stored at –80C.

**Antibodies.**

P-AKT substrate (RXXS*/T*) (9614S, 1:1000), AKT (pan) (4691S, 1:1000), P-AKT (S473) (4060L, 1:1000), P-PRAS40 (T246) (2997S, 1:1000), PRAS40 (tot) (2691S, 1:1000), vinculin (12901S, 1:1000), p-p70 S6 Kinase (T389) (9234S, 1:1000), p70 S6 Kinase (2708S, 1:1000), PERK (3192S, 1:1000), BiP/GRP78 (3177S, 1:1000), P-EGFR (Y1068) (3777S, 1:1000), P-EGFR (Y845) (D63B4, 1:1000), EGF Receptor (4267S, 1:1000), PD-L1 (13684S, 1:1000), HER3 (12708S, 1:1000), E-Cadherin (3195S, 1:1000), GST (2625S, 1:1000), and HA-Tag (2367S, 1:1000) were purchased from Cell Signaling Technology. ALG3-1 (Q92685_1, 1:300) was custom made by GenScript. B-Actin (067M4856V, 1:1000) was purchased from Sigma. The ALG3-1 antibody generated by GenScript was validated by siRNA-mediated depletion of endogenous ALG3 using a pooled siRNA from Dharmacon. Specificity was further confirmed by loss of signal in ALG3 CRISPR knockout cells and detection of tagged ALG3 in overexpression and immunoprecipitation experiments.

**Lectins.**

Galanthus Nivalis Lectin (GNL/GNA), Biotinylated (B-1245-2, 1:750), Concanavalin A (Con A), Biotinylated (B-1005-5, 1:1500), Phaseolus Vulgaris Erythroagglutinin (PHA-E) , (B-1125-2, 1:750), Sambucus Nigra Lectin (SNA, EBL), Biotinylated (B-1305-2, 1:750), Griffonia (Bandeiraea) Simplicifolia lectin I (GSL-I), (BK-2100, 1:750), Ricinus Communis Agglutinin (RCA-I), Biotinylated (BK-1000, 1:750), and Ulex Europaeus Agglutinin (UEA-I), Biotinylated (BK-1000, 1:750) were all purchased from Vector Laboratories.

**Drug Treatment Experiments**

For signaling experiments cells were plated in 15 cm plates. Two days later, cells are serum/growth-factor-deprived (20 hours). Cells are then pre-treated for 15 minutes with DMSO or small-molecule inhibitors (GDC0941, GDC0068, or Rapamycin), then treated with insulin for the indicated time period. Cells were then harvested as described in ‘Immunoprecipitation analysis of Endogenous ALG3’.

**Immunoblotting**

Cells were washed with 1X PBS (Boston Bioproducts) and then either flash frozen and stored at –80C until lysis, or lysed fresh, using radioimmunoprecipitation buffer (RIPA) (1% Nonidet P-40, 0.5% sodium deoxycholate, 0.1% SDS, 150 mM NaCl, 50 mM Tris-HCl, pH 7.5, protease inhibitor mixture, 50 nM calyculin A, 1mM sodium pyrophosphate, and 20mM sodium fluoride) for 15 minutes. Cell extracts were precleared by centrifugation at 14,000 rpm for 10 min at 4C, and then cleared by a second centrifugation at 14,000 rpm for 10 min at 4C. The Bio-Rad DC protein assay was used to measure the protein concentration, and then sample concentration was normalized using RIPA and SDS sample buffer. All samples in which ALG3-1 is detected are not boiled or frozen and loaded fresh. All other samples are boiled for 5 min at 95C. Lysates were resolved on acrylamide gels by SDS-polyacrylamide gel electrophoresis and electrophoretically transferred to nitrocellulose membrane (BioRad) at 300 mA for 90 minutes at 4C in Tris-glycine transfer buffer (Boston BioProducts). Membranes were blocked in TBS buffer (10 mmol/L Tris-HCl, pH 8, 150 mmol/L NaCl; Boston Bioproducts) containing 5% (w/v) nonfat dry milk (Andwin Scientific). Membranes were incubated with specific primary antibodies diluted in 5% (w/v) fatty-acid-free bovine serum albumin (BSA) (Boston BioProducts, P-753) in TBS-T with 0.01% (w/v) sodium azide (Thermo Fisher Scientific) and rocking at 4°C overnight. Membranes were washed in TBST at room temperature three times for 10 minutes and if developed in the dark room were incubated in horseradish peroxidase (HRP)-conjugated secondary antibody diluted in 5% (w/v) milk in TBS-T for 1 hour, with rocking at room temperature. The membrane was washed again with TBS-T then incubated in Clarity Western ECL Substrate (Bio-Rad, 1705061) or Clarity MAX Western ECL Substrate (Bio-Rad, 1705062), then developed. If the membrane was not incubated with HRP secondary, it was incubated in fluorophore-conjugated secondary antibodies (LI-COR Biosciences) diluted in 5% (w/v) milk in TBS-T for 1 hour, with rocking at room temperature. The membrane was washed again with TBS-T then the chemiluminescence signal was imaged with a Li-Cor Odyssey CLx Imaging System (LI-COR Biosciences).

**Immunoprecipitation analysis of endogenous ALG3.**

All steps were carried out on wet ice or at 4°C. Cells were lysed from 15 cm dishes in 100 ul of RIPA buffer (1% Nonidet P-40, 0.5% sodium deoxycholate, 0.1% SDS, 150 mM NaCl, 50 mM Tris-HCl, pH 7.5, protease inhibitor mixture, 50 nM calyculin A, 1mM sodium pyrophosphate, and 20mM sodium fluoride) on ice for 15 min. Lysates were centrifuged at 14,000 rpm for preclearing for 10 min at 4°C, the supernatants transferred to a new tube, then centrifuged a second time at 14,000 rpm for 10 minutes at 4°C, and the cleared supernatants were transferred to a new tube. The Bio-Rad DC protein assay was used to assess protein concentration, and sample concentrations were normalized using RIPA and SDS sample buffer. 1.4 mg of lysate was incubated for 2 hours with immunoprecipitation antibody, rocking at 4°C. Immunoprecipitation antibodies were bound to Protein A/G agarose beads that had been washed three times with PBS, then resuspended in a 1:1 slurry in RIPA. Additional lysate was saved for immunoblotting the whole cell lysate. Then, 20 ul of resuspended beads were added to each immunoprecipitation sample for an additional 2 hours with rocking at 4°C. Three washes were performed in RIPA lysis buffer and beads were centrifuged at 2,500g, followed by removal of all of the supernatant. One wash was performed using PBS. The samples were then resuspended in 1X sample buffer and heated to 65°C for 20 minutes to release the protein from the beads, then loaded on acrylamide gels by SDS-polyacrylamide gel electrophoresis. Samples are not boiled or frozen.

**Immunoprecipitation analysis of endogenous EGFR.**

All steps were carried out on wet ice or at 4°C. Cells were lysed from 15 cm dishes in 100 ul of NP-40 buffer (40mM Hepes pH7.4, 120mM NaCl, 1mM EDTA pH 8.0, 1% NP-40 (CA-630), 5% glycerol, protease inhibitor mixture, 10mM pyrophosphate (sodium pyrophosphate tetrabasic), 10mM b-glycerophosphate (glycerol 2-phosphate), 50mM NaF in ddH2O) on ice for 15 min. Lysates were centrifuged at 14,000 rpm for preclearing for 10 min at 4°C, the supernatants transferred to a new tube, then centrifuged a second time at 14,000 rpm for 10 minutes at 4°C, and the cleared supernatants were transferred to a new tube. The Bio-Rad DC protein assay was used to assess protein concentration, and sample concentrations were normalized using NP-40 and SDS sample buffer. 1.4 mg of lysate was incubated for 2 hours with immunoprecipitation antibody [15 ul of 10ug/mL EGF Receptor (4267S)], rocking at 4°C. Immunoprecipitation antibodies were bound to Protein A agarose beads that had been washed three times with PBS, then resuspended in a 1:1 slurry in NP-40. Additional lysate was saved for immunoblotting the whole cell lysate. Then, 20 ul of resuspended beads were added to each immunoprecipitation sample for an additional 2 hours with rocking at 4°C. Three washes were performed in NP-40 lysis buffer and beads were magnetically bound to a column after washes, followed by removal of all of the supernatant. Between washes, samples were rocked at 4°C during the wash. The samples were then resuspended in 1X sample buffer and heated to 65°C for 20 minutes to release the protein from the beads, then loaded on acrylamide gels by SDS-polyacrylamide gel electrophoresis. Samples were not boiled or frozen.

**Immunoprecipitation analysis of MSCV-HA-FLAG-ALG3.**

Samples were lysed as described in the ‘Immunoprecipitation analysis of endogenous ALG3’ section. Anti-flag M2 agarose beads (Millipore-Sigma, A2220) were added to lysates and then samples were rocked for 2 hours at 4°C to immunopurify HA-FLAG-ALG3. The beads were washed three times in full RIPA lysis buffer, and one time with 1X PBS at 4°C; beads were centrifuged at 14,000 rpm, followed by removal of all of the supernatant. Excess buffer was removed from above the settled beads before resuspended in 1X sample buffer and heated to 65°C for 20 minutes for release of protein from the beads then loaded on acrylamide gels by SDS-polyacrylamide gel electrophoresis. Samples are not boiled or frozen. After transfer, gels were Coomassie stained, and then incubated for 1 hour with specific primary antibodies diluted in 5% (w/v) fatty-acid-free bovine serum albumin (BSA) (Boston BioProducts, P-753) in TBS-T with 0.01% (w/v) sodium azide (Thermo Fisher Scientific) and rocking at 4°C overnight. Membranes were washed in TBST at room temperature three times for 10 minutes and if developed in the dark room were incubated in horseradish peroxidase (HRP)-conjugated secondary antibody diluted in 5% (w/v) milk in TBS-T for 1 hour, with rocking at room temperature. The membrane was washed again with TBS-T then incubated in Clarity Western ECL Substrate (Bio-Rad, 1705061) or Clarity MAX Western ECL Substrate (Bio-Rad, 1705062) then developed in the dark room.

**Growth Rate Assay.**

Cell lines were plated at densities ranging from 5,000-25,000 cells per well in 24-well plates and allowed 24 hours to adhere before a day zero reading is taken. Population size as cell confluency was assayed using sulforhodamine B staining, as described in (Vichai V., and Kirtikara K. (2006) Sulforhodamine B colorimetric assay for cytotoxicity screening. Nat. Protoc. 1, 1112–1116 10.1038/nprot.2006.179). Cells were fixed in 8.3% final concentration of tricarboxylic acid for at least 1 hour at 4°C at the indicated day. Cells were then washed three times with DI water. Cells were then stained for at least 30 min with 0.5% sulforhodamine B in 1% acetic acid. Cells were then washed three times with 1% acetic acid and then the sulforhodamine B dye was solubilized in 10mM Tris pH 10.5. Absorbance was read at 510 nm using the BioTek Synergy LX multi-mode plate reader. Relative growth was determined as (growth at each day/growth at day 0).

**Lentivirus production.**

293T cells were plated in 10cm dishes at 9x10^6^ cells/plate then transiently transfected with 6.3 ug of the plasmid of interest to generate lentivirus. 11.1 ug of the psPAX2 packaging plasmid (Addgene plasmid, 12260, created by the laboratory of D. Trono), 0.6 ug of pCMV-VSV-G envelope protein plasmid (Addgene plasmid 8454, created by the laboratory of B. Weinberg) and 8.6 ul of polyethylenamine (Millipore-Sigma, 408727) per ug of DNA transfected. After 48 hours, media is collected, passed through a 0.45 um filter (Thermo Fisher Scientific, 09-740-106), aliquoted to 5 mL aliquots, and stored at –80°C and only thawed for use once, never re-frozen.

**Retrovirus production.**

293T cells were plated in 10 cm dishes at 9x10^6^ cells/plate then transiently transfected with 2.5 ug of the plasmid of interest to generate retrovirus. 1.5 ug of the pCL-Eco packaging plasmid (Addgene plasmid, 12260, created by the laboratory of D. Trono), 1 ug of pCMV-VSV-G envelope protein plasmid (Addgene plasmid 8454, created by the laboratory of B. Weinberg) and 21.6 ul of polyethylenamine (Millipore-Sigma, 408727) per ug of DNA transfected. After 48 hours, media is collected, passed through a 0.45 um filter (Thermo Fisher Scientific, 09-740-106), aliquoted to 5 mL aliquots, and stored at –80°C and only thawed for use once, never re-frozen.

**ALG3 CRISPR-Cas9 Knockouts*.***

The pLentiCRISPRv2-Blast vector was used, which would constitutively express the sgRNA and the blasticidin resistance marker (Addgene plasmid, 83480 created by the laboratory of Mohan Babu), containing two separate single guide RNA (sgRNA) sequences targeting exon 5 (sgALG3_2) and exon 4 (sgALG3_3).The sgRNAs were designed using Benchling. Guide oligos were synthesized by Integrated DNA Technologies and inserted into the vector as previously described (Genome-scale CRISPR-Cas9 knockout screening in human cells Science 2014). The plasmids were transformed into NEB Stable competent high-efficiency Escherichia coli (New England Biolabs, C3040H) then sequenced. Lentivirus was produced as described in ‘Lentivirus production’. Cells were infected with lentivirus for 48 hours, then virus was removed and cells were selected with blasticidin (Invivogen, ant-bl-1) for 5-7 days until uninfected cells were completely dead from treatment. SgRNA sequences: CACACAGTTTGGCTTCCGTG (ALG3_2), GCGGCTCTTCAATGACCCAG (ALG3_3)

**Site-directed mutagenesis.**

The site-directed mutagenesis of the phosphorylation sites on ALG3 as well as the site-directed mutagenesis of the sequence where guide sgALG3_3 binds (to generate a guide-resistant version of the vector) were performed using self-designed primers with the support of Benchling and using the QuickChange II XL Site-Directed Mutagenesis Kit (Agilent , 200521). The guide-resistant vector reaction site-directed mutagenesis was designed to change the G at base 2038 on the vector so the sequence would be changed from TGCGGCTCTTCAATGACCCAG to TGCGCCTCTTCAATGACCCAG. The forward primer used is: 5' CATCTTTGTGCTGCGCCTCTTCAATGACCC 3' and the reverse primer is: 5' GGGTCATTGAAGAGGCGCAGCACAAAGATG 3'. This single silent mutation is sufficient to stop CRISPR-Cas9-mediated cutting of the ALG3 protein (confirmed with testing by western blotting and qRT-PCR).

**MSCV-N-Flag-HA-IRES-PURO-ALG3 expression constructs.**

The full-length human ALG3 sequence with a stop codon at the end was inserted into N-terminally HA (YPYDVPDYA) and Flag-tagged (DYKDDDDK) MSCV construct. An guide-resistant version of the MSCV-N-Flag-HA-IRES-PURO_ALG3 construct was also generated by site-directed mutagenesis to be resistant to guide sgALG3_3. Phosphomutant versions of ALG3 (Ser11Ala, Ser13Ala, Ser11Ala/Ser13Ala) were also inserted into the N-terminally HA and Flag-tagged MSCV construct and additionally, sgALG3_3-resistant phosphomutant lines were generated. Empty MSCV-N-Flag-HA-IRES-PURO was used as a control for transfections and experiments. These expression constructs were transfected using HEK293T cells to produce retrovirus that was used to infect cells (see 'Retrovirus production’).

**Generation of cell lines with stable expression.**

Cells were plated in 10 cm plates at 1x6^6^ cells/plate. Lentivirus/retrovirus was added with 5 ug ml^-1^ polybrene (Sigma-Aldrich, 107689) to infect cells the next day. Lentivirus/retrovirus was left on the cells for 48 hours, then removed and cells were split into selection media containing 1 ug ml^-1^ puromycin (MSCV) or 5-7.5 ug ml^-1^ Blasticidin (pLentiCRISPR). Cells were selected until uninfected cells containing the same dosage of puromycin/blasticidin were completely dead.

**Lectin Blotting Analysis.**

Glycoprotein samples were prepared as described in ‘Immunoblots’ identically through the transfer step. After transfer, membranes were blocked in 5% (w/v) fatty-acid-free bovine serum albumin (BSA) (Boston BioProducts, P-753) in TBS (ThermoFisher Scientific) overnight. The blocking buffer was removed, and membranes were then incubated with biotinylated lectin diluted in 5% w/v BSA for 30 minutes rocking at room temperature. The lectin was removed, and the membranes were washed 3 times, for 10 minutes each with TBS-T (ThermoFisher Scientific) rocking at room temperature. Washing buffer was removed and Streptavidin-HRP or Streptavidin-DyLight-800 diluted in 5% w/v BSA in TBS for 30 minutes rocking at room temperature. The membranes were then washed 3 times for 10 minutes each with TBS-T rocking at room temperature. If Streptavidin-HRP was used, membranes were then incubated with Clarity Western ECL Substrate (Bio-Rad, 1705061) or Clarity MAX Western ECL Substrate (Bio-Rad, 1705062), then developed in the dark room. If Streptavidin-DyLight-800 was used, membranes were then imaged with a Li-Cor Odyssey CLx Imaging System (LI-COR Biosciences).

***In Vitro* Kinase Assays.**

MCF10A cells were plated at 5x10^5^ cells/plate in 10A plates, The next day media was removed, cells were washed with serum/growth-factor-free media, then serum starved for 20 hours. Serum-free media was then replenished for 2 hours then cells were lysed on ice and rocked for 10 minutes at 4C. Cell extracts were precleared by centrifugation at 14,000 rpm for 10 min at 4C, and then cleared by a second centrifugation at 14,000 rpm for 10 min at 4°C. The Bio-Rad DC protein assay was used to measure the protein concentration, and then 2 mg of protein was immunoprecipitated, and the rest of the cell lysate saved for whole cell lysate western blotting. 30 ul of GLAF beads were added and samples were rotated for 2 hours at 4C. Samples were spin at 8K xg at 4°C for 1 minute, and then supernatant was removed. Samples were washed 4 times: 1 ml NETN buffer was added, the tube was inverted 5 times and incubated on ice for 2 min, then spun at 8K xg at 4°C for 1 min and supernatant removed. Tubes were washed 1 time with 1X PBS. A mastermix of reaction mix minus enzyme was prepared for each sample using water, Kinase assay buffer (10X) (cell signaling #9802): with freshly added DTT (final 2 mM), ATP (10 mM) (Cell signaling #9804, or home-made), and Akt1, active (Sigma SRP5001). 45 ul of reaction mix minus enzyme was added to each tube with 5 ul of GST-AKT1, tubes were mixed gently by flicking, then incubated for 1 hour at 30C. Beads were flicked every 10 minutes during this hour. Reaction was stopped by adding 12 ul 6X SDS loading buffer and samples were boiled at 65C for 20 minutes and then spun at 8K x g for 30 seconds, then loaded (30 or 35 ul) on a gel for electrophoresis and subsequent western blotting.

**Quantitative real-time PCR.**

Total RNA was isolated with the NucleoSpin RNA Plus (MACHEREY-NAGEL) according to the manufacturer’s protocol. Reverse transcription was performed using the SuperScript III First-Strand Synthesis System (ThermoFisher Scientific) using random hexamers as the primer. A CFX384 Touch Real-Time PCR Detection System (BioRad) was used to perform the quantitative real-time PCR (qRT-PCR). 18S rRNA was used as the reference gene when in calculations to quantify mRNA expression. ALG3 primers were designed using Benchling. BiP/GRP78^1,2^, CHOP^1,2^, HER3^2^, EGFR^2,4^, and E-Cadherin^2,5^ primers have been published.

Primer Sequences (F=Forward, R=Reverse):

ALG3-1-F: 5’GGGACTCTGCAAGCAATGGC3’

ALG3-1-R: 5’ACCCTGTGAATGACCCAGAAGG3’

ALG3-2-F: 5’GGGACTCTGCAAGCAATGGC3’

ALG3-2-R: 5’CCACCCTGTGAATGACCCAGAA3’

ALG3-3-F: 5’CCTACATGGCCGAGGTAGAAGG3’

ALG3-3-R: 5’ACAAGTGGTCCGGTGTCACC3’

ALG3-4-F: 5’TGGGGTTGTACTATGCCACCAG3’

ALG3-4-R: 5’GAAGACAAGCAGCAAGGTAGCC3’

ALG3-5-F: 5’CGTGTCCACTCCATCTTTGTGC3’

ALG3-5-R: 5’GCCAGCAGGAGGTTGATACTGA3’
BiP-1-F: 5’CTGTCCAGGCTGGTGTGCTCT3’.

BiP-1-R: 5’CTTGGTAGGCACCACTGTGTTC3’

BiP-2-F: 5’TCTGGTACTGCTTGATGTTTGTC3’.

BiP-2-R: 5’GTCCGCATCCTGGTGGCTTTCC3’

BiP-3-F: 5’TGGGTCGACTCGAATTCCAAAG3’

BiP-3-R: 5’ GTCAGGCGATTCTGGTCATTGG

CHOP-1-F: 5’GGTATGAGGACCTGCAAGAGGT3’

CHOP-1-R: 5’ CTTGTGACCTCTGCTGGTTCTG3’

CHOP-2-F: 5’TGCCTTTCACCTTGGAGAC3’’

CHOP-2-R: 5’ CGTTTCCTGGGGATGAGATA3’

HER3-F: 5’CTATGAGGCGATACTTGGAACGG3’

HER3-R: 5’GCACAGTTCCAAAGACACCCGA3’

EGFR-F: 5’AACACCCTGGTCTGGAAGTACG3’

EGFR-R: 5’TCGTTGGACAGCCTTCAAGACC3’

E-Cad-F: 5’GCCTCCTGAAAAGAGAGTGGAAG3’

E-Cad-R: 5’TGGCAGTGTCTCTCCAAATCCG3’

18S-F: 5’-CTTAGAGGGACAAGTGGCG-3’

18S-R: 5’-ACGCTGAGCCAGTCAGTGTA-3’

**General Chemistry Methods.**

Reagents and solvents were purchased from commercial suppliers and were used without further purification unless otherwise noted.

**Summary Table for Vector Descriptions, Labels, and Simplified Labels in the Manuscript**

| **Label** | **Description** | | **Simplified Label** |
| --- | --- | --- | --- |
| MSCV-HA-FLAG-ALG3-WT | | Wild-type ALG3 overexpression construct with HA and Flag tags on the N-terminus | ALG3-WT |
| MSCV-HA-FLAG-ALG3-WT-SG3R | | Wild-type ALG3 overexpression construct with HA and Flag tags on the N-terminus; resistant to sgALG3_3 (through site-directed mutagenesis base change) | ALG3-WT-SG3R |
| MSCV-HA-FLAG-S11A-SG3R | | ALG3 with Ser11 mutated to alanine; resistant to sgALG3_3 (through site-directed mutagenesis base change) | ALG3-S11A |
| MSCV-HA-FLAG-S13A-SG3R | | ALG3 with Ser13 mutated to alanine; resistant to sgALG3_3 (through site-directed mutagenesis base change) | ALG3-S13A |
| MSCV-HA-FLAG-S11/13A-SG3R | | ALG3 with Ser11 and Ser13 mutated to alanine; resistant to sgALG3_3 (through site-directed mutagenesis base change) | ALG3-S11/13A |
| MSCV-HA-FLAG-EV | | Empty vector control for the MSCV-HA-FLAG overexpression vector | EV |
| pLENTI_CRISPR_BLAST_EV | | Empty vector control for the pLENTI-CRISPR-BLAST vector containing guide RNA | EV |
| pLENTI_CRISPR_BLAST_sgALG3_2 | | pLENTI-CRISPR-BLAST construct expressing CRISPR guide RNA targeting ALG3 (site 2). Used for CRISPR/Cas9-mediated knockout of endogenous ALG3. | sgALG3_2 |
| pLENTI_CRISPR_BLAST_sgALG3_3 | | pLENTI-CRISPR-BLAST construct expressing CRISPR guide RNA targeting ALG3 (site 3). Used for CRISPR/Cas9-mediated knockout of endogenous ALG3. | sgALG3_3 |

**Replication and Statistical Analysis**

Proliferation assays were conducted three independent times (three biological replicates), each with three technical replicates. Proliferation data are displayed as one biological replicate with corresponding technical replicates, as representative data. Proliferation data were averaged and normalized to day 0 time point. Statistical analyses for proliferation data were performed with PRISM 10 graphing software (GraphPad). Each data point represents the mean +/- SD. Statistical analyses for SRB growth curves were performed by completing a one-way ANOVA at the latest time point.

qRT-PCR experiments were conducted with 2-4 biological replicates per condition, each with at least two technical replicates. The data shown in each figure include a minimum of three technical replicates per condition. Bar graphs represent the mean +/- SD. Details are outlined below.

Immunoblot experiments were completed with at least two independent biological replicates per condition; representative blots from one biological replicate are shown. Only experiments with confirmed knockout efficiency were used for analysis. We distinguish between the number of independent times an experiment was completed with biological replicates, and representative data are shown in figure panels. A detailed breakdown of the number of biological and technical replicates completed in the lab versus those shown in each figure panel is provided to ensure clarity and transparency.

**Summary of Replicates for Each Figure Panel:**

Fig 1A – Phosphosite graphic – N/A

Fig 1B – Motif Alignment – N/A

Fig 1C – The displayed immunoblot is from a single experiment (N=1) and is representative of three independent biological replicates conducted.

Fig 1D – The displayed immunoblot is from a single experiment (N=1) and is representative of three independent biological replicates.

Fig 1E – The displayed immunoblot is from a single experiment (N=1) and is representative of three independent biological replicates.

Fig 1F – The displayed immunoblot is from a single experiment (N=1) and is representative of three independent biological replicates.

Fig 2A – The displayed qRT-PCR data are from a single biological replicate with four technical replicates. Specifically, two distinct primer pairs were used, each run in duplicate, for a total of four technical replicates. The result is representative of seven independent biological replicates performed throughout the course of the study.

Fig 2B – The displayed immunoblot is from a single experiment (N=1) and is representative of three independent biological replicates.

Fig 2C – The displayed immunoblot is from a single experiment (N=1) and is representative of three independent biological replicates.

Fig 2D – The displayed immunoblot is from a single experiment (N=1) and is representative of three independent biological replicates.

Fig 2E – The displayed immunoblot is from a single experiment (N=1) and is representative of two independent biological replicates.

Fig 3A – The displayed immunoblot is from a single experiment (N=1) and is representative of three independent biological replicates.

Fig 3B – The displayed qRT-PCR is from a single biological replicate. For *GRP78*, three distinct primer pairs were used, each run in duplicate, for a total of six technical replicates. For *CHOP*, two distinct primer pairs were used, each run in duplicate, for a total of four technical replicates. The result is representative of three independent biological replicates.

Fig 3C – The displayed immunoblot is from a single experiment (N=1) and is representative of three independent biological replicates.

Fig 3D – The displayed immunoblot is from a single experiment (N=1) and is representative of one independent biological replicate.

Fig 3E – The displayed qRT-PCR is from two biological replicates, each with two technical replicates (total of four replicates shown on the graph). The result is representative of two independent biological replicates.

Fig 4A – The displayed proliferation data for the parental and EV lines are from two biological replicates, each with three technical replicates (six replicates total). The displayed proliferation data for the sgALG3_2 and sgALG3_3 cell lines are from a single biological replicates, each with three technical replicates. The variability in replicate numbers reflects the combination of normalized data from two independent experiments. The results are representative of a total of three independent biological replicates per CRISPR guide to ALG3, each of which included three technical replicates.

Fig 4B – The displayed proliferation data are from one biological replicate with three technical replicates. The results are representative of a total of six independent biological replicates.

Fig 4C – The displayed proliferation data are from one biological replicate with 18 technical replicates. The results are representative of a total of three independent biological replicates.

Fig 4D – The displayed proliferation data are from one biological replicate with three technical replicates. The results are representative of a total of three independent biological replicates.

Fig 4E – The displayed proliferation data is from one biological replicate with three technical replicates. The results are representative of a total of three independent biological replicates.

Fig 4F – The displayed immunoblot is from a single experiment (N=1) and is representative of two independent biological replicates.

**References**

1. Roh, J.-I. *et al.* Impaired AKT signaling and lung tumorigenesis by PIERCE1 ablation in KRAS-mutant non-small cell lung cancer. *Oncogene* **39**, 5876–5887 (2020).

2. OriGene Technologies Inc. QSTAR qPCR Primer Pairs. https://www.origene.com/products/gene-expression/qpcr/primer-pairs.

3. Banik, D. *et al.* HDAC6 plays a noncanonical role in the regulation of antitumor immune responses, dissemination, and invasiveness of breast cancer. *Cancer Res.* **80**, 3649–3662 (2020).

4. Fan, J.-B. *et al.* microRNA-7 inhibition protects human osteoblasts from dexamethasone via activation of epidermal growth factor receptor signaling. *Mol. Cell. Biochem.* **460**, 113–121 (2019).

5. Duan, F. *et al.* Loss of GFAT1 promotes epithelial-to-mesenchymal transition and predicts unfavorable prognosis in gastric cancer. *Oncotarget* **7**, 38427–38439 (2016).
